# Supplementary material for: Content-rich biological network constructed by mining PubMed abstracts
Source: BMC Bioinformatics. 2004 Oct 8;5:147. doi: 10.1186/1471-2105-5-147 (PMC528731; doi:10.1186/1471-2105-5-147)
Supplement: Additional File 5 — The original Chilibot query results of the term "long-term potentiation (LTP)" and 22 other terms, limiting the latest references analyzed to the years 1990, 1995, 2000, and 2004. [file 1471-2105-5-147-S5.bz2 › chilibotAdditionalFile5/ltp2004/html/NMDA_ATF.html]

 


 **NMDA** and **ATF** 
  
Found 1 abstracts in PubMed,  **1 abstracts were retrieved and analyzed**.  


---

 Search Google  |
 PDF files only 
|  EDU domain only 

---

- Rev Neurol< MedlineTA>Rev Neurol,   Cell signaling in the epileptic hippocampus Cell signaling commanding death or survival in human epileptic hippocampus is difficult to trace because of the long interval between the beginning of symptoms and the sampling of damaged cerebral tissue for neuropathological examination.
  Intraperitoneal injection of the glutamate analogue kainic acid is a useful tool to analyze the effects of seizures and the excitotoxic damage in the rodent hippocampus.
  KA acts on **NMDA** and KA receptors.
  . . . it has little impact on AMPA receptors.
  Neurons of the hilus and CA3 neurons are primary targets of KA, although parvalbumin containing GABAergic neurons are less vulnerable than glutamatergic neurons.
  Immediate responses to KA are hsp 70 mRNA induction and HSP 70 72 protein expression, as well as c fos and c jun mRNA, and c Fos and c Jun protein expression in the hippocampus.
  Yet increased c Fos and c Jun expression is not a predictor of cell death or cell survival.
  In contrast, the tissular plasminogen activator and the membrane Fas Fas L signaling pathway probably have a role in facilitating cell death following KA injection.
  The involvement of other pathways remains controversial.
  Increased expression of the pro apoptotic Bax together with decreased Bcl 2 suggests Bax mediated apoptosis.
  Activation of the mitochondrial pathway includes leakage of citochrome c to the cytosol and activation of the caspase cascade leading to apoptosis.
  However, other studies have emphasized the limited expression of caspase 3, the main executioner of apoptosis, and the relevance of necrosis as the main form of cell death following KA excitotoxicity.
  Phosphorylation dependent activation of several kinases, including MAPK, p 38 and JNK SAPK, and their substrates has been found in KA treated animals.
  Decreased CREBp expression is associated with cell death whereas increased **ATF** 2P and Elk 1P are associated with cell survival.
  Trophic factors probably do not play a significant role during the early stages of hippocanmpal damage but they are important in the remodeling of the granukle cells and the sprouting of mossy fibers to the molecular layer of the dentate gyrus.
  This abnormal regeneration, in turn, facilitates seizure recruitment and the chronic maintenance of convulsions.
